# Supplementary material for: Texture Feature Extraction from 1H NMR Spectra for the Geographical Origin Traceability of Chinese Yam
Source: Foods. 2023 Jun 24;12(13):2476. doi: 10.3390/foods12132476 (PMC10340326; doi:10.3390/foods12132476)
Supplement: Supplementary file 1 [file foods-12-02476-s001.zip › foods-2438525-supplementary.pdf]

Supplementary Table

Table S1. Samples of geo-authentic Chinese yam

Index	Producing areas	Harvest site(E, N)	Harvest and experiment time	Soil texture	Number
WL	Wenxian	112.990, 35.005	2019.11	clay soil	11
WS	Wenxian	113.135, 34.924	2019.11	sandy soil	10
ML	Mengzhou	112.902, 34.918	2019.11	clay soil	9
MS	Mengzhou	112.884, 34.873	2019.11	sandy soil	10
WZL	Wuzhi	113.180, 34.988	2019.11	clay soil	10
WZS	Wuzhi	113.309, 34.985	2019.11	sandy soil	10
QL	Qinyang	112.941, 35.026	2019.11	clay soil	10

Table S2. Samples of non-authentic Chinese yam

Index	Producing areas	Harvest site(E, N)	Harvest and experiment time	Number	Soil texture
XBZ	Qingyuan County	115.56, 38.62	2017.11	10	clay soil
ZY	Gaoyang County	115.75, 38.67	2017.11	10	clay soil
CJ	Chenji Town	115.41, 35.12	2017.11	10	clay soil
JPC	Chenji Town	113.37, 34.44	2017.11	10	clay soil
XS	Chenji Town	113.37, 34.44	2017.11	10	clay soil

WX	Wenxi County	111.33, 35.46	2017.11	10	clay soil
YJ	Yongji County	110.28, 34.90	2017.11	10	clay soil
